# Supplementary material for: A NIR-Activated and Mild-Temperature-Sensitive Nanoplatform with an HSP90 Inhibitor for Combinatory Chemotherapy and Mild Photothermal Therapy in Cancel Cells
Source: Pharmaceutics. 2023 Aug 31;15(9):2252. doi: 10.3390/pharmaceutics15092252 (PMC10537501; doi:10.3390/pharmaceutics15092252)
Supplement: Supplementary file 1 [file pharmaceutics-15-02252-s001.zip › pharmaceutics-2455687-supplementary.pdf]

pharmaceutics

MDPI

Supplementary Material: A NIR-Activated and Mild-Temperature-Sensitive Nanoplatform with HSP90 Inhibitor for Combinatory Chemo- and Mild Photothermal Therapy in Cancel Cells

Yingying Peng, Hanlin Jiang, Bifei Li, Yue Liu, Bing Guo and Wei Gan

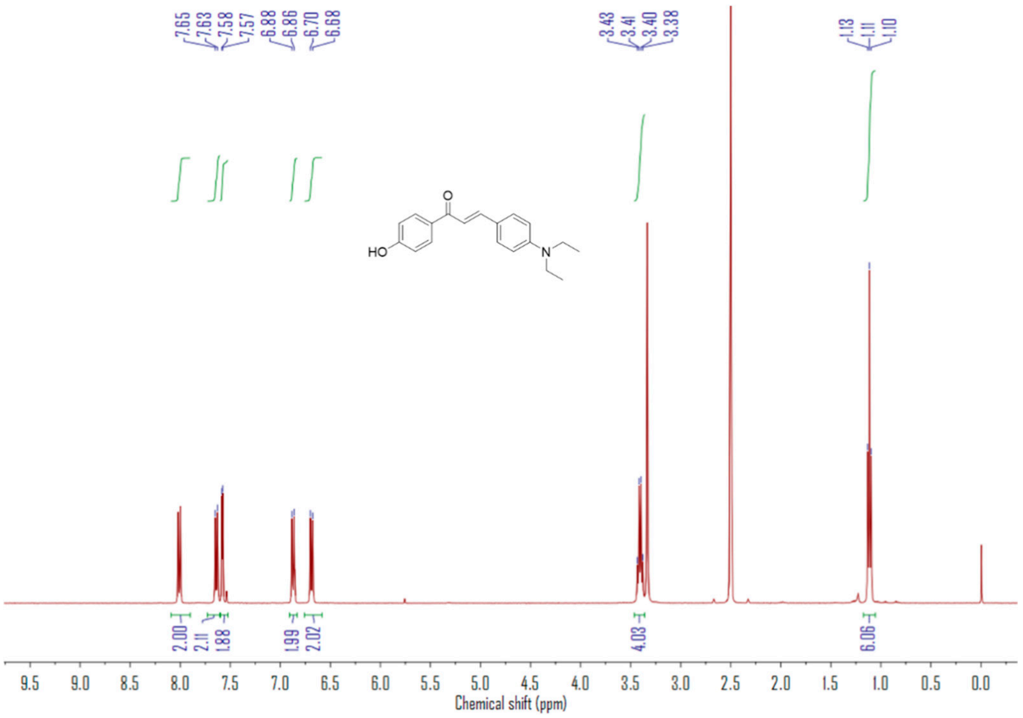

Figure S1. The ^{1}H NMR spectrum of compound **3** in DMSO-*d6*.

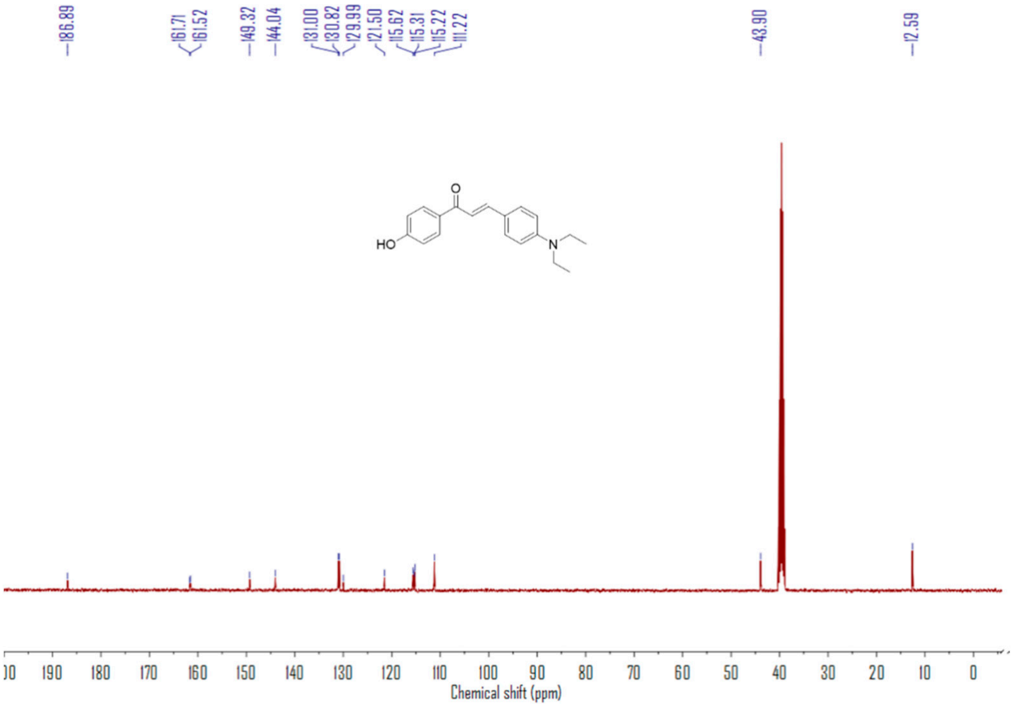

Figure S2. The ^{13}C NMR spectrum of compound **3** in DMSO-*d6*.

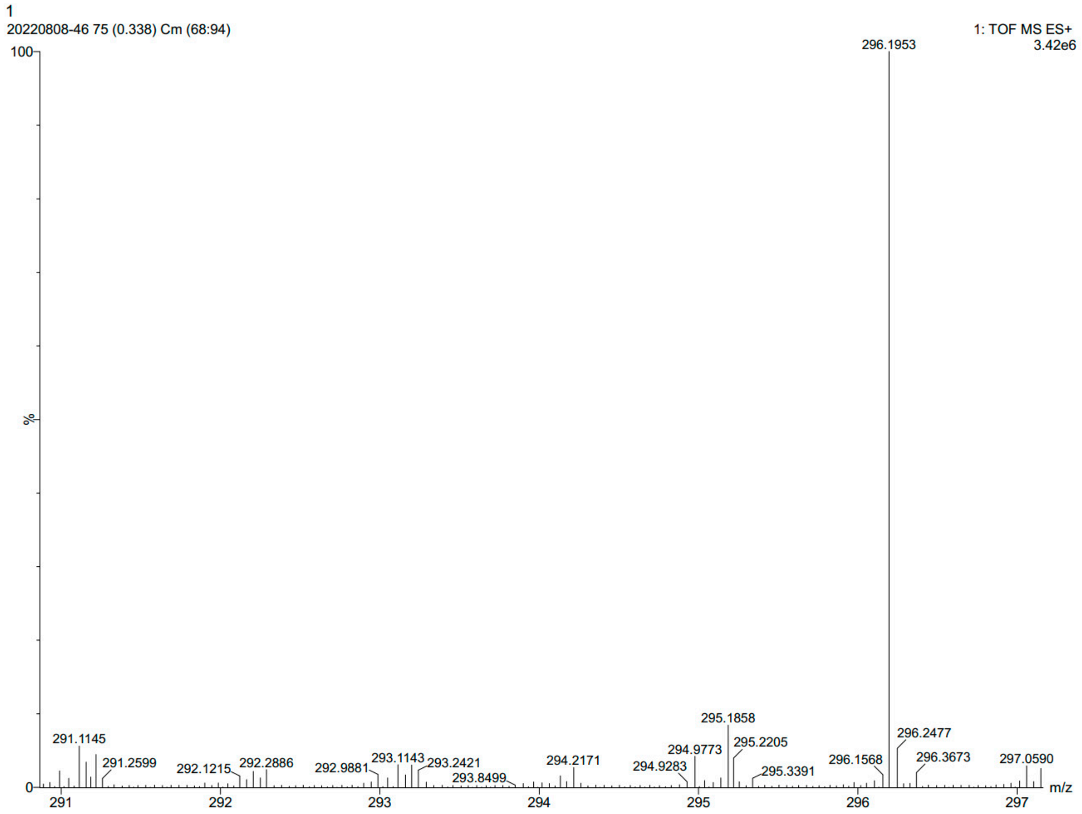

Figure S3. ESI-Mass spectrum of compound **3**.

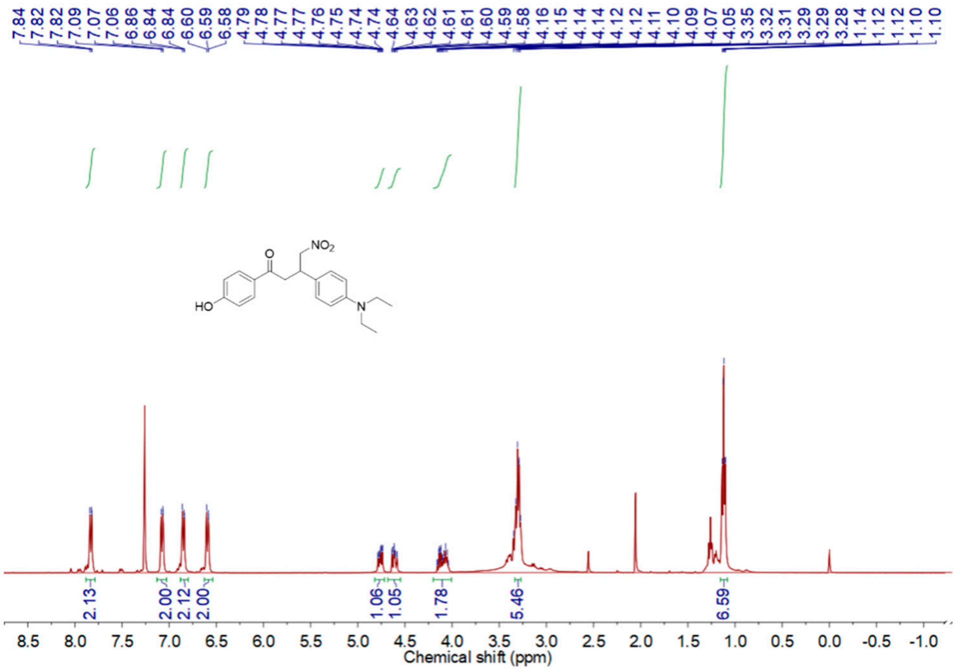

Figure S4. The ^{1}H NMR spectrum of compound **4** in $CDCl_3$.

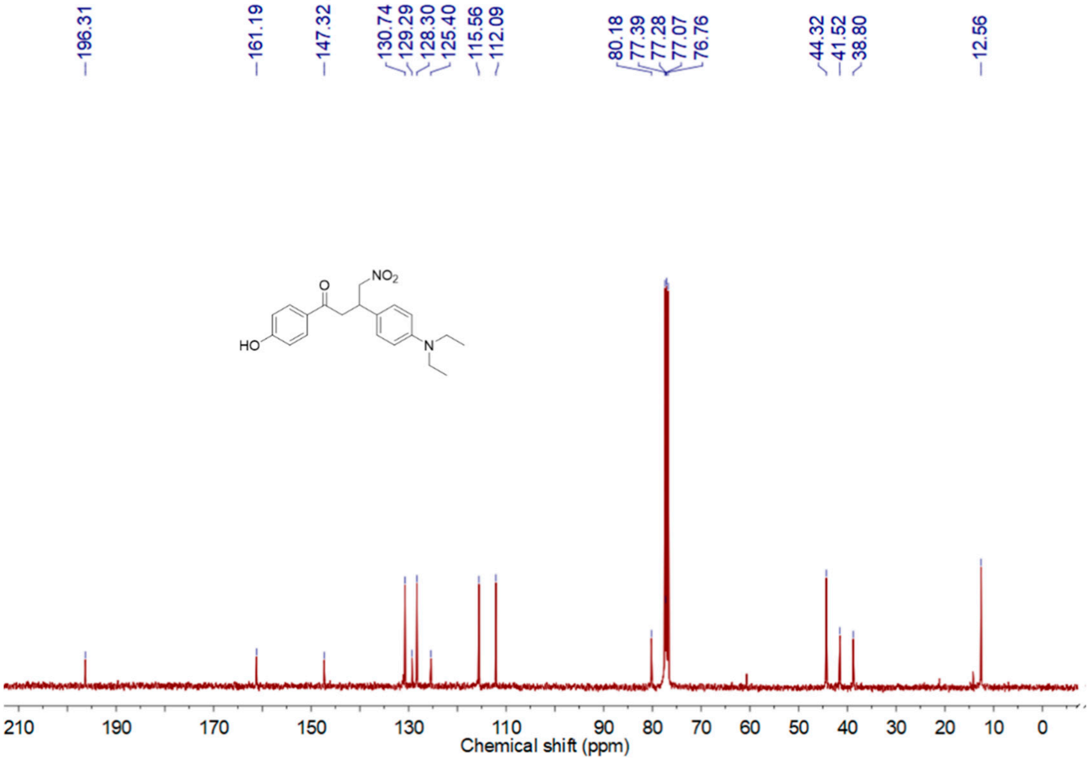

Figure S5. The ^{13}C NMR spectrum of compound **4** in $CDCl_3$.

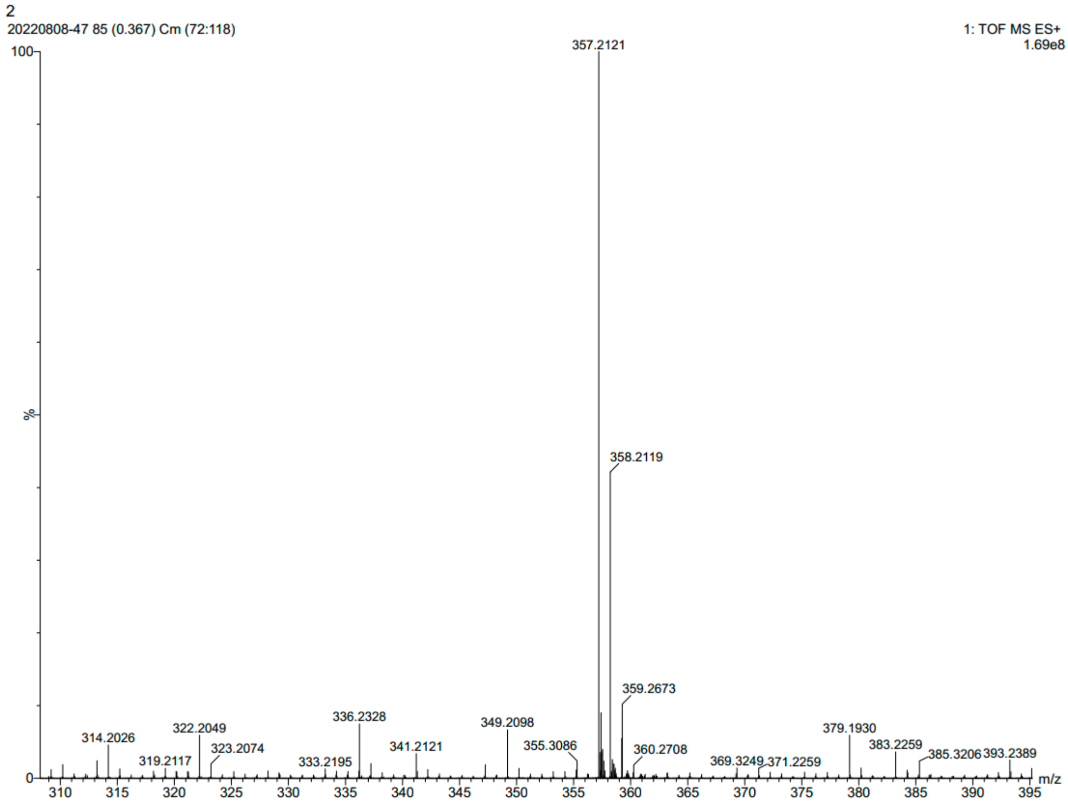

Figure S6. ESI-Mass spectrum of compound **4**.

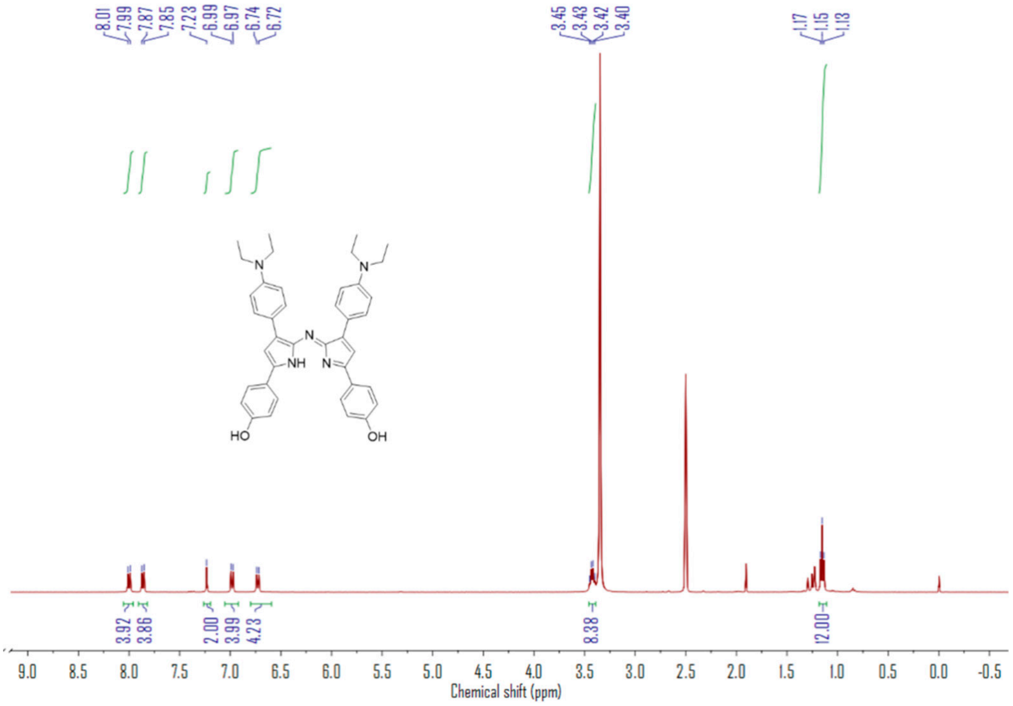

Figure S7. The ^{1}H NMR spectrum of compound **5** in DMSO-*d6*.

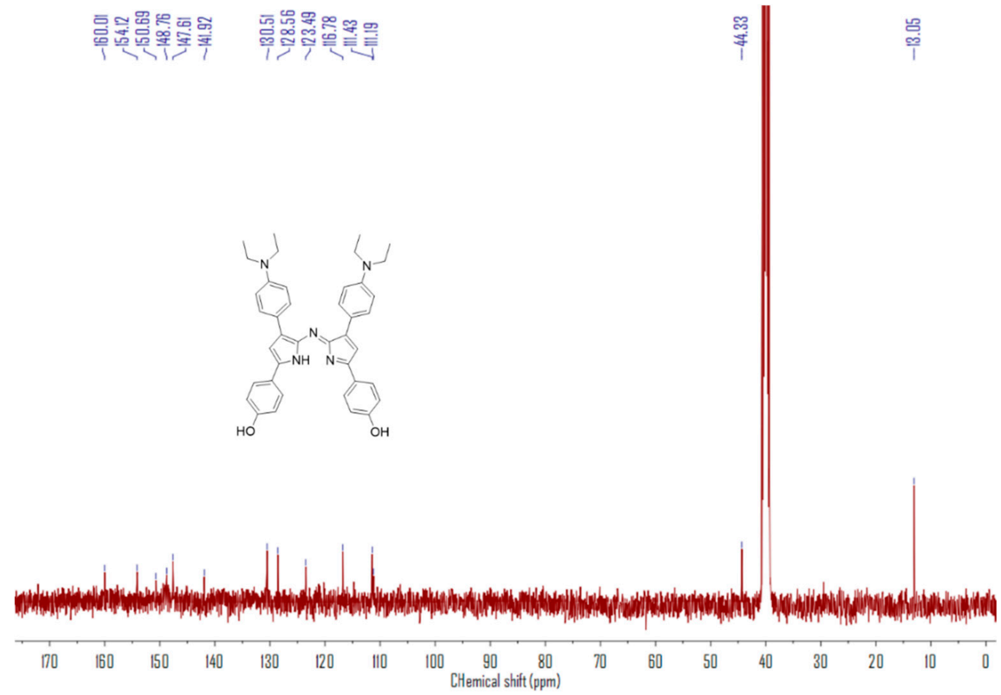

Figure S8. The ^{13}C NMR spectrum of compound **5** in DMSO-*d6*.

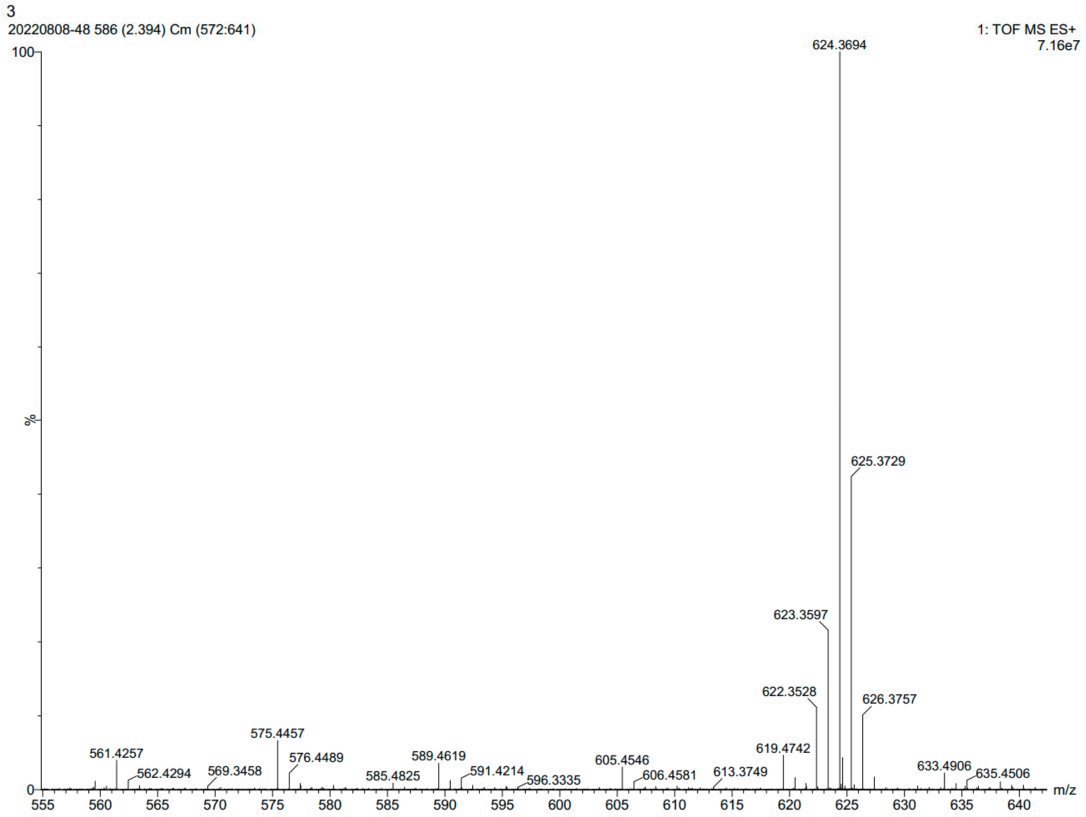

Figure S9. ESI-Mass spectrum of compound **5**.

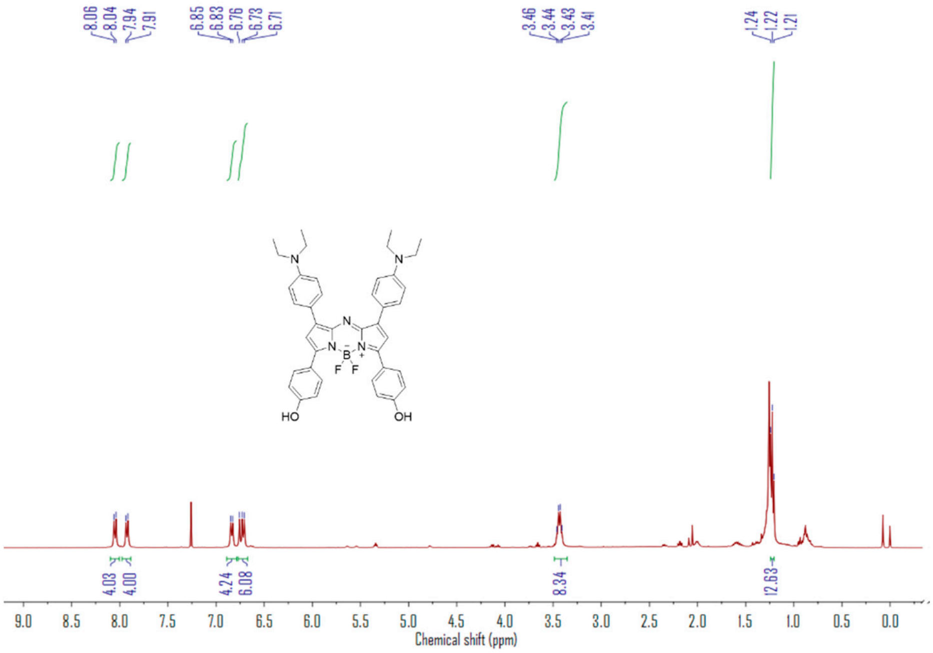

Figure S10. The ^{1}H NMR spectrum of **BDPII** in $CDCl_3$.

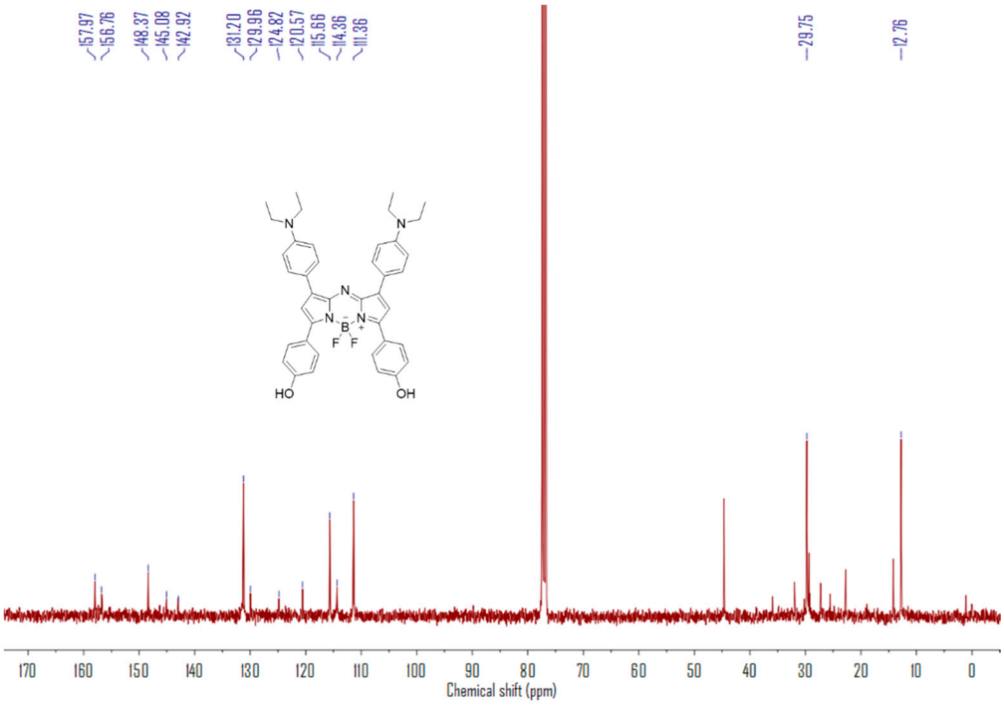

Figure S11. The ^{13}C NMR spectrum of **BDPII** in $CDCl_3$.

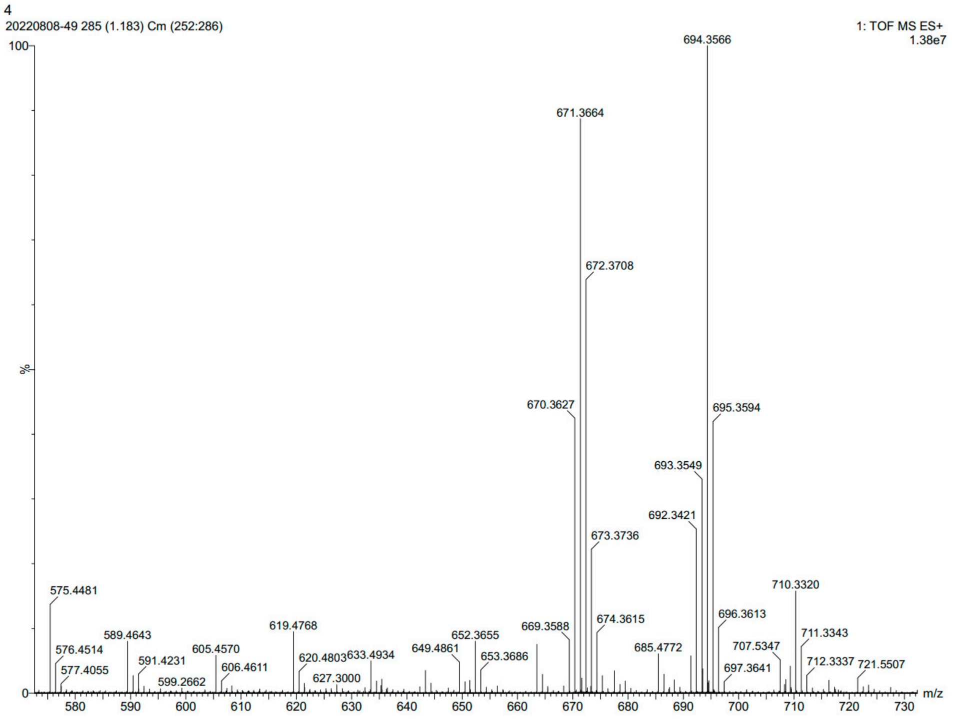

Figure S12. ESI-Mass spectrum of **BDPII**.

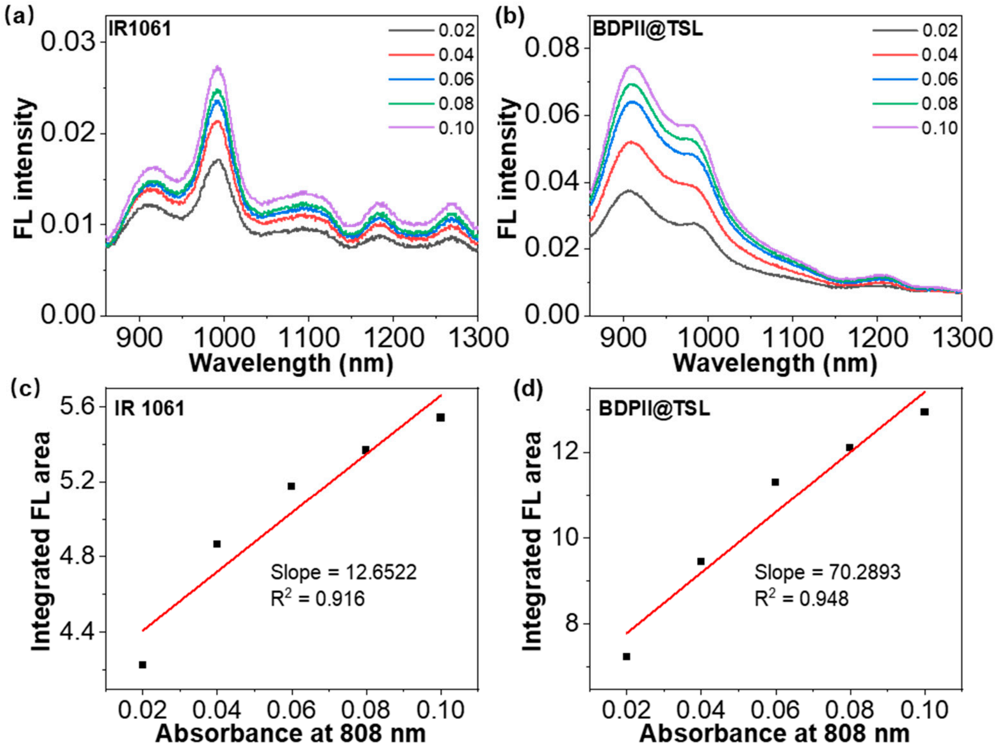

Figure S13. The emission spectra of the (a) IR1061 solution and (b) BDPII@TSL NPs with different concentration upon 808 nm excitation (Legend: the absorbance at 808 nm for the corresponding sample solution). The integrated fluorescence emission area at 900-1300 nm as a function of absorbance at 808 nm of (c) IR1061 and (d) BDPII@TSL solutions.

Table S1. The average diameter of BDPII@TSL and BDPII-gel@TSL in water, PBS and DMEM with 4 °C storage for 15 days.

		**In Water**		
Compound	1st	5th	10th	15th
BDPII@TSL	117±1.63 nm	11±0.86 nm	115±3.32 nm	112±1.28 nm
BDPII-gel@TSL	139±0.34 nm	139±1.24 nm	134±2.20 nm	1±1.98 nm
		**In PBS**		
Compound	1st	5th	10th	15th
BDPII@TSL	135±1.69 nm	138±1.30 nm	145±2.58 nm	144±0.74 nm
BDPII-gel@TSL	138±1.25 nm	137±0.62 nm	136±2.31 nm	136±2.12 nm
		**In DMEM**		
Compound	1st	5th	10th	15th
BDPII@TSL	155±1.81 nm	156±2.06 nm	153±1.17 nm	153±1.62 nm
BDPII-gel@TSL	137±1.45 nm	140±1.17 nm	152±1.34 nm	154±1.49 nm

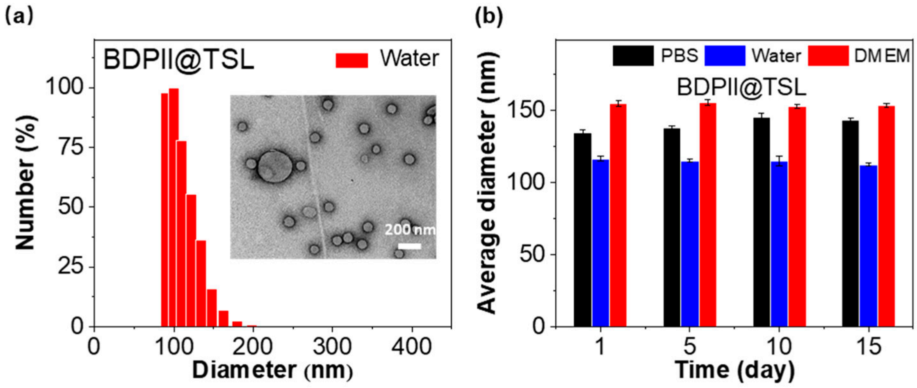

Figure S14. (a) The size distribution of BDPII@TSL NPs measured by dynamic light scattering as well as the particles morphology for BDPII@TSL NPs detected by transmission electron microscopy (inner) with a white scale bar of 200 nm. (b) The average diameter of BDPII@TSL NPs in water, PBS and DMEM with 4 °C storage for 15 days.

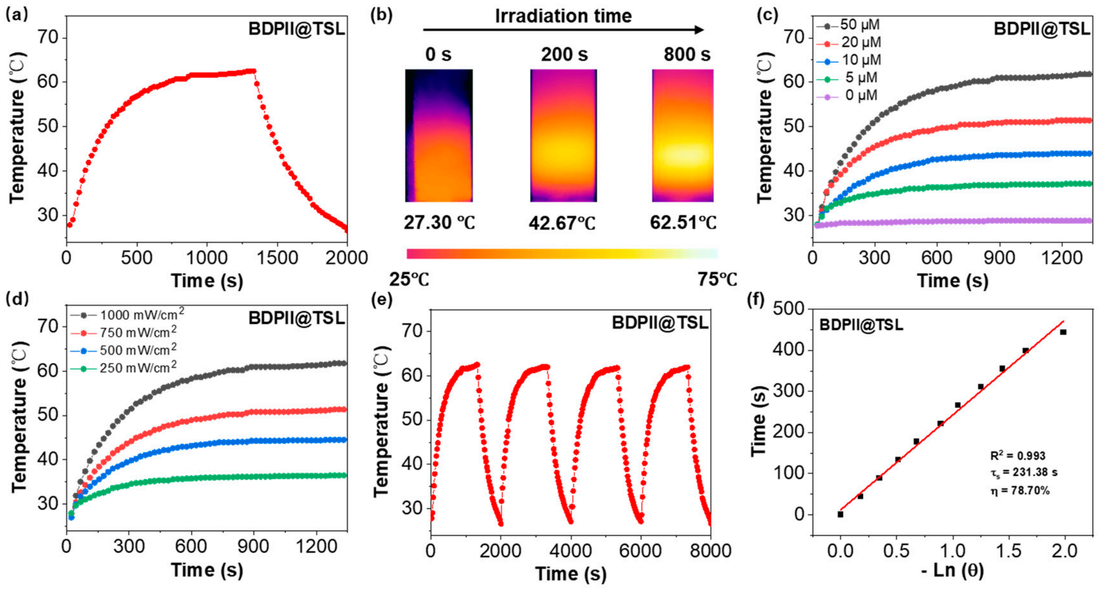

Figure S15. (a) Photothermal heating effect for BDPII@TSL in water (40 μM) upon 808 nm irradiation with 1 W cm^{-2} laser power followed by the cooling process to room temperature. (b) The infrared photos for BDPII@TSL NPs at different irradiation time (1 W cm^{-2}, 808 nm). The photothermal heating profiles for BDPII@TSL NPs with different (c) concentration (1 W cm^{-2}, 808 nm) and (d) laser power density (40 μM, 808 nm). (e) Photothermal stability for BDII@TSL NPs (40 μM) upon 808 nm irradiation with laser power of 1 W cm^{-2} for four switching on/off cycles. (f) The plot of the negative natural logarithm of driving force temperature versus the cooling time for BDPII@TSL in the photothermal heating process.

Table S2. The average fluorescence intensity for HeLa cells incubated with calcein-AM (green)/PI (red) and no particles (control), geldanamycin, BDPII@TSL and BDPII-gel@TSL NPs without and with 808 nm irradiation for 30 min.

		**With light**		
Compound	control	geldanamycin	BDPII@TSL	BDPII-gel@TSL
green	148.09	107.87	32.54	5.98
red	0.34	46.67	98.79	139.68
Ratio (red/green)	0.00	0.43	3.04	23.36
		**Without light**		
Compound	control	geldanamycin	BDPII@TSL	BDPII-gel@TSL
green	147.98	96.14	149.33	151.28
red	0.12	39.87	1.20	21.98
Ratio (red/green)	0.00	0.41	0.00	0.15
